# Antibacterial activity of silver nanoparticles: sensitivity of different *Salmonella* serovars

**DOI:** 10.3389/fmicb.2014.00227

**Published:** 2014-05-26

**Authors:** Carmen Losasso, Simone Belluco, Veronica Cibin, Paola Zavagnin, Ivan Mičetić, Federica Gallocchio, Michela Zanella, Lisa Bregoli, Giancarlo Biancotto, Antonia Ricci

**Affiliations:** ^1^Department of Food Safety, Istituto Zooprofilattico Sperimentale delle VenezieLegnaro, Italy; ^2^European Center for the Sustainable Impact of Nanotechnology, Veneto Nanotech S.C.p.A.Rovigo, Italy

**Keywords:** *Salmonella*, antimicrobials, silver resistance, nanoparticles, silver

## Abstract

*Salmonella* spp. is one of the main causes of foodborne illnesses in humans worldwide. Consequently, great interest exists in reducing its impact on human health by lowering its prevalence in the food chain. Antimicrobial formulations in the form of nanoparticles exert bactericidal action due to their enhanced reactivity resultant from their high surface/volume ratio. Silver nanoparticles (AgNPs) are known to be highly toxic to Gram-negative and Gram-positive microorganisms, including multidrug resistant bacteria. However, few data concerning their success against different *Salmonella* serovars are available. Aims of the present study were to test the antimicrobial effectiveness of AgNPs, against *Salmonella* Enteritidis, Hadar, and Senftenberg, and to investigate the causes of their different survival abilities from a molecular point of view. Results showed an immediate, time-limited and serovar-dependent reduction of bacterial viability. In the case of *S*. Senftenberg, the reduction in numbers was observed for up to 4 h of incubation in the presence of 200 mg/l of AgNPs; on the contrary, *S.* Enteritidis and *S.* Hadar resulted to be inhibited for up to 48 h. Reverse transcription and polymerase chain reaction experiments demonstrated the constitutive expression of the plasmidic silver resistance determinant (SilB) by *S.* Senftenberg, thus suggesting the importance of a cautious use of AgNPs.

## INTRODUCTION

*Salmonella* spp. is recognized as one of the main causes of foodborne illnesses in humans worldwide. In 2011, a total of 95,548 confirmed cases of human salmonellosis were reported in the EU. Moreover, this microorganism is responsible for the largest number of reported European food-borne outbreaks (26.6% of all outbreaks; EFSA and ECDC, 2013). Two species belong to the genus *Salmonella*: *enterica* and *bongori*. *S. enterica* includes six subspecies and more than 2500 serovars, based on the Kauffmann–White Le Minor scheme (Grimont and Weill, 2007), with different features in terms of resistance, infectivity, morbidity, and mortality. Even though *Salmonella* Enteritidis and Typhimurium are the two most frequently isolated serovars among humans (Foley et al., 2011), any serovar is considered capable of causing gastrointestinal illness of varying severity in humans.

Following these considerations, *Salmonella* nowadays represents a major challenge in animal health and food safety (EFSA and ECDC, 2013); consequently great interest exists in reducing its impact in human health by lowering its prevalence in the food chain through a farm to fork approach. Different control measures have proven to be effective against *Salmonella* at farm level, such as vaccination and proper hygiene management, whereas the use of antimicrobials is forbidden according to EU Regulation 1177/2006, due to the risk of spread of antimicrobial resistance (COMMISSION REGULATION (EC) No 1177/2006 of 1 August 2006, 2005).

Even though EU Regulations on *Salmonella* control at farm level focus the attention on a limited number of serovars, Regulation EC 2073/2005 on microbiological criteria for foodstuffs (COMMISSION REGULATION (EC) No 2073/2005 of 15 November 2005 on microbiological criteria for foodstuffs, 2006) compels food business operators to ensure the absence of *Salmonella* spp. in some foods of animal origin. In cases of non-compliance with the food safety criteria set out in the Regulation, the products shall be withdrawn or recalled, which naturally poses serious problems for food producers.

For these reasons, it is interesting to investigate the efficacy and applicability of new types of safe and effective biocidal compounds. Silver has been used for centuries as an antimicrobial (Silver et al., 2006) to fight infections and prevent spoilage (Rai et al., 2009), and it is well known that silver ions and silver-based compounds are highly toxic to Gram-negative and Gram-positive microorganisms (Bae et al., 2010; Fayaz et al., 2010; You et al., 2011; Maillard and Hartemann, 2012). Previous studies have shown that antimicrobial formulations in the form of nanoparticles (NPs) could be used as effective bactericidal materials due to their enhanced reactivity, resulting from their high surface/volume ratio (Pal et al., 2007; Choi et al., 2008; Park et al., 2009). Particularly, silver in the form of NP (AgNP) is known to exhibit strong biocidal effects on different bacterial species (Sondi and Salopek-Sondi, 2004; Rai et al., 2009), including multidrug resistant bacteria (Lara et al., 2010). It is generally accepted that free silver ions, present or released from the nanomaterials, are able to bind cell membrane structures, destabilizing the membrane potential and causing proton leakage (Gogoi et al., 2006; Maillard and Hartemann, 2012). Even though the effectiveness of AgNPs has been tested against the genus *Salmonella* (Chiao et al., 2012), no data concerning different *Salmonella* serovars including those which are of the greatest concern to humans, are available to date. Furthermore, the use of silver as an antimicrobial has been found to select resistance determinants (Pelgrift and Friedman, 2013), but little information is available on the possibility that the same effect could also be exerted by AgNPs (Samberg et al., 2011). Moreover, the presence of silver-resistance genes in environmental *Salmonella* isolates needs to be established (Maillard and Hartemann, 2012) and the resistance to AgNPs of silver resistant bacteria should be confirmed. In fact, NPs have different physiochemical properties, compared to those of bulk material of the same composition, which may possibly result in different toxicity mechanism(s) in biological systems (Nel et al., 2006). The exponential increase in the use of AgNPs in recent years raises some concerns about the risk associated with potential resistance in the bacterial community (Maillard and Hartemann, 2012). Silver resistance in food chain-associated bacteria could be a potential public health problem since silver is a key component in several products (Gupta et al., 1999) specifically devoted to food contact materials. However, data arising from scientific studies on the biological activity of NPs are difficult to interpret due to the peculiar chemical and physical characteristics of these particles. Thus, it is fundamental to provide detailed characterization of any tested NPs, as this would allow the scientific community to compare particles more rationally.

Aims of the present study were to test the antimicrobial effectiveness of silver in the form of NPs (AgNPs) against different *Salmonella* serovars (Enteritidis, Hadar, Senftenberg) and to investigate the causes of their different survival ability, when identified, from a molecular point of view.

## MATERIALS AND METHODS

### NANOPARTICLES AND CHEMICALS

Silver nanoparticles suspensions (code NM-300K batch n. 6051) and their control dispersant medium were purchased from LGC Standards (UK). These NPs are included in the Joint Research Center Nanomaterials Repository, are approved within the OECD WPMN (Working Party on Manufactured Nanomaterials) international testing program as selected representative reference nanomaterial (OECD Paris, 2010), and are delivered in aqueous solution containing 4% of polyoxyethylene glycerol trioleate and polyoxyethylene sorbitan mono-laurate (Tween 20). Silver nitrate (AgNO_3_) was purchased from Sigma-Aldrich (USA).

### BACTERIAL STRAINS

Bacterial wild strains, *S.* Enteritidis (3546/6 2012) isolated from an environmental swab of a layer hens’ pen, *S.* Hadar (2507/5 2009) isolated from a broiler chicken cloacal swab and *S.* Senftenberg (3014/3 2012) isolated from an environmental swab of a layer hens’ pen, were obtained from the collection of pathogenic microorganisms of the OIE Reference Laboratory for Salmonellosis (Istituto Zooprofilattico Sperimentale delle Venezie, Legnaro, Italy). *Salmonella* strains were stored in Microbank^TM^ (Pro-Lab Diagnostics, USA) at -80°C until needed. *Klebsiella pneumoniae* clone ST258 (García-Fernández et al., 2012), used as positive control, was kindly provided by Dr Alessandra Carattoli at Istituto Superiore di Sanità, Rome, Italy. The stock cultures were subcultured on tryptone agar (TA) slants at 4°C, and *Salmonella* serovars were confirmed by serotyping according to Grimont and Weill (2007), transferred to 15 ml of Mueller-Hinton broth (MHB) and incubated at 37°C overnight.

### ANTIMICROBIAL SUSCEPTIBILITY TESTING

*Salmonella* strains were tested for susceptibility to antimicrobials (**Table 1**) using a commercial microdilution test (Sensititre^®^ *Salmonella* plate – EUMVS2) according to the manufacturer’s recommendations. The results were read visually after 24 h of incubation at 37°C, and the minimum inhibitory concentration (MIC) was defined as the lowest concentration of the antimicrobial that completely inhibited visible growth.

**Table 1:** *Salmonella* serovars used and their susceptibility to antibiotics, described as minimum inhibitory concentration (MIC mg/l).

MIC (mg/l)	*S.* Enteritidis	*S.* Hadar	*S.* Senftenberg
Sulfamethoxazole	64	>1.024	>1.024
Gentamicin	0.50	1	0.50
Ciprofloxacin	0.015	0.5	0.25
Ampicillin	0.5	>32	0.25
Cefotaxime	0.06	0.12	0.12
Ceftazidime	0.25	0.50	0.25
Tetracycline	1	32	1
Streptomycin	2	64	16
Trimethoprim	0.5	0.5	0.5
Chloramphenicol	4	8	8
Colistin	2	2	2
Florfenicol	4	4	8
Kanamycin	4	4	4
Nalidixic acid	4	>64	>64

### AgNPs CHEMICAL PURITY

Chemical purity of AgNPs was evaluated by inductively coupled plasma mass spectrometry (ICP-MS, NexION 300D PerkinElmer). In order to obtain a suitable concentration for ICP-MS analysis, a diluted solution of AgNPs was prepared (105 mg/kg), then microwave-assisted digestion (Mars5, CEM) was performed on triplicate samples (average weight: 0.200 g) according to the method suggested by Klein et al. (2011). Accurate control of contamination was performed by processing four method blanks. A semi-quantitative analysis was carried out to gain a general evaluation of the impurities, and then the most relevant elements were quantified by an external calibration method.

### SIZE AND MORPHOLOGY OF AgNPs

AgNPs morphology, size and stability over time in the culture medium, were analyzed by transmission electron microscopy (TEM) and dynamic light scattering (DLS). TEM analysis was performed with a Fei Tecnai 12 G2 microscope operated at 120 kV. NPs were suspended in ultrapure water at 100 mg/l, deposited on carbon coated copper grids and left to dry overnight. Micrographs were recorded on a side mounted Olympus Morada CCD camera. Size distribution analysis was performed using a semi-automatic method implemented in ImageJ software (Schneider et al., 2012). The analysis involved particle segmentation and area determination. The diameter of each particle was estimated from the particle area assuming a perfectly circular particle. For DLS analyses, AgNPs 50 mg/l suspension in ultrapure water was analyzed with a Malvern Zetasizer Nano ZS DLS instrument operated in backscattering mode. Measurements were carried out at 23°C and were conducted in triplicate to check for sedimentation and solution stability.

### AgNPs SUSCEPTIBILITY ASSAY

The effectiveness of AgNPs as an antimicrobial was determined by assaying the number of culturable bacterial cells which formed colonies after incubation in the presence of AgNPs or AgNO_3_. Silver nitrate was used to investigate the effect of silver ions. MHB was used as culture medium with the objective of obtaining a good compromise between *Salmonella* spp. growth and NPs stability, given that this is a quite simple and non-selective liquid medium, specifically designed for susceptibility studies and having the minimum requirements for bacteria growth.

Briefly, overnight *Salmonella* inocula, grown in MHB to an OD A_600_ = 0.3 [10^8^ colony-forming units (CFUs)/ml], were incubated in an orbital shaker at 200 rpm at 37°C for 30 min, 1, 2, 4, 24, 48, and 72 h in the presence of AgNPs (200, 150, 100, 50, 20 mg/l) or AgNO_3_ (20, 15, 10 mg/l). Two controls were included: one flask containing NPs and nutrient media devoid of inoculum, and one flask containing inoculum and nutrient media, devoid of NPs or silver nitrate.

The high rotary shaking speed was selected to minimize the aggregation and settlement of the NPs over the incubation period. At the indicated time points (30 min, 1, 2, 4, 24, 48, and 72 h) 100 μl aliquots of serial 10-fold dilutions of bacterial cultures in MHB were plated on xylose lysine tergitol 4 (XLT4) agar plates. Colonies were counted after incubation at 37°C for 24 h and counts expressed as CFU/ml. All counts were checked also after 48 h in order to exclude the presence of colonies possibly sub-lethally injured (but not killed) by AgNPs.

Each of the experiments was performed in triplicate and the CFU counts were normalized and then summarized as mean values and standard deviations (SDs). For statistical purposes a value of 300 colonies was assigned to plates on which more than 300 colonies were observed (according to ISO 7218/2007). The AgNPs and AgNO_3_ concentrations causing inhibitory effects were determined based on the absence of colonies on the agar plates.

### SILVER RESISTANCE MOLECULAR TESTING

Bacterial total DNA was extracted by boiling extraction method. Briefly, one single colony of each serovar growing on solid media was resuspended in 100 μl of sterile molecular biology-grade water (Eppendorf, Hamburg, Germany) in a microcentrifuge tube, subjected to boiling at 100°C for 10 min, cooled on ice, and centrifuged at 15,000 *g* for 10 s before being stored at -20°C. Total DNA was quantified spectrophotometrically by using a SmartSpec^TM^ Plus spectrophotometer (Bio-Rad Life Science, USA) and aliquoted.

The plasmidic DNA fraction was extracted from pelleted cells using the QIAprep Spin Miniprep Kit (Qiagen). The concentration and purity of plasmid DNA was determined spectrophotometrically by measuring the A260nm/A280nm ratio.

### REVERSE TRANSCRIPTION AND POLYMERASE CHAIN REACTION

Overnight *S.* Senftenberg inocula, grown on MHB to an OD A_600_ = 0.3 (10^8^ CFU/ml), were incubated in an orbital shaker at 200 rpm at 37°C for 4 and 24 h in the presence of AgNPs (0 and 200 mg/l) or AgNO_3_ (0 and 20 mg/l). Cell cultures were pelleted and total RNA isolated using the NucliSENS^®^ kit (Biomérieux, Italia SPA). The concentration and purity of RNA was determined spectrophotometrically by measuring the A260nm/A280nm ratio.

For cDNA synthesis, the Enhanced Avian reverse transcription and polymerase chain reaction (RT-PCR) kit (Sigma-Aldrich, USA) was used. Total RNA (5 μg) was incubated in DEPC-treated water with 2.5 μM oligodT, preheated at 70°C for 10 min, and incubated in the presence of avian reverse transcriptase at 42°C for 1 h following the conditions recommended by the manufacturer.

PCR samples were prepared by adding 5 μl of 4 ng/μl DNA template to a reaction mix containing 200 μM dNTPs, 0.4 μM of each primer, 1× polymerase buffer, 2 mM MgCl_2_ and 1.5 U of AmpliTaq Gold (Applied Biosystem, USA) DNA polymerase; the reaction volume was made up to 50 μl with nuclease-free water. PCRs were carried out in a GeneAmp PCR System 9700 (Applied Biosystem, USA) using the following PCR protocol: 5 min at 95°C followed by 30 cycles of 95°C for 30s, 57°C for 30s, and 72°C for 30 s. A final elongation step was performed at 72°C for 5 min. SilB gene primers sequences, SilB forward (CAAAGAACAGCGCGTGATTA) and SilB reverse (GCTCAGACATTGCTGGCATA), were designed according to Woods et al. (2009).

PCR products were resolved in 1% agarose Tris acetate-EDTA (TAE) gel, stained with ethidium bromide (Sigma, Dorset, UK), electrophoresed for 1 h at 70 V/cm and visualized with a Bio-Rad Gel doc system. A Perfect DNA Markers (EMD, Millipore, USA), 0.05–10 kb ladder was applied to the gel to define PCR fragment sizes.

## RESULTS

### AgNPs CHARACTERIZATION

**FIGURE 1:**
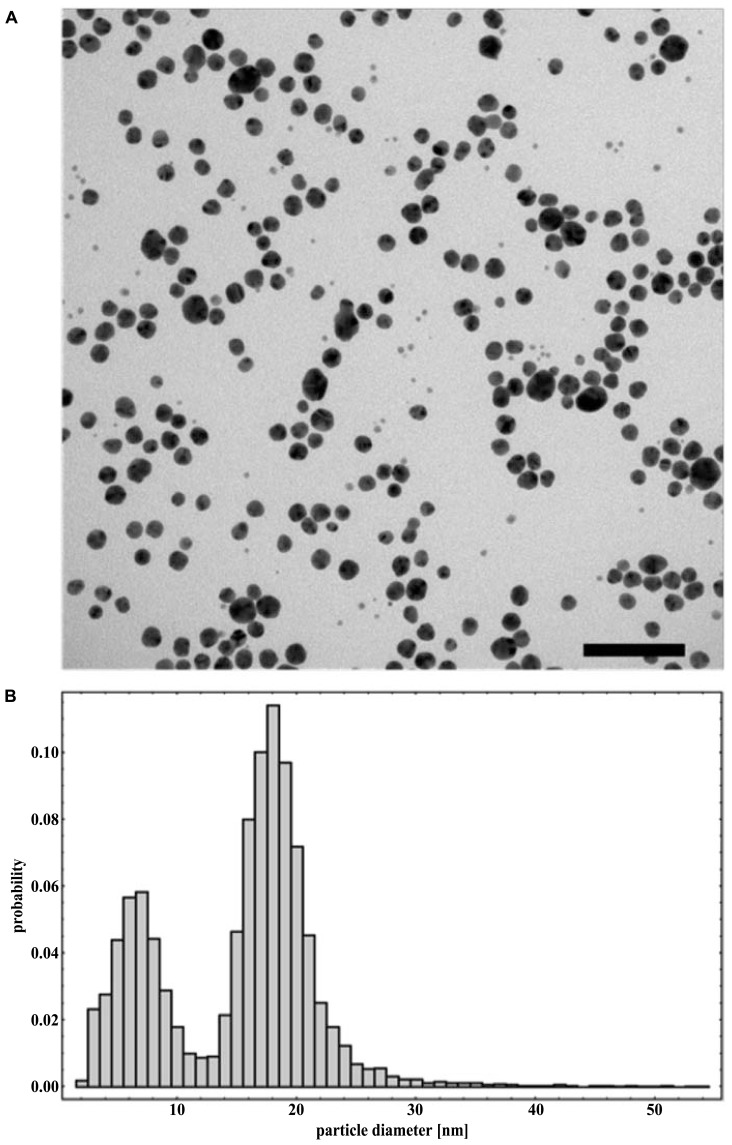
**Representative TEM micrograph (A) and particle size distribution obtained from TEM data (B) of NM-300K silver nanoparticles dispersed in water.** Scale bar equals 100 nm.

**FIGURE 2:**
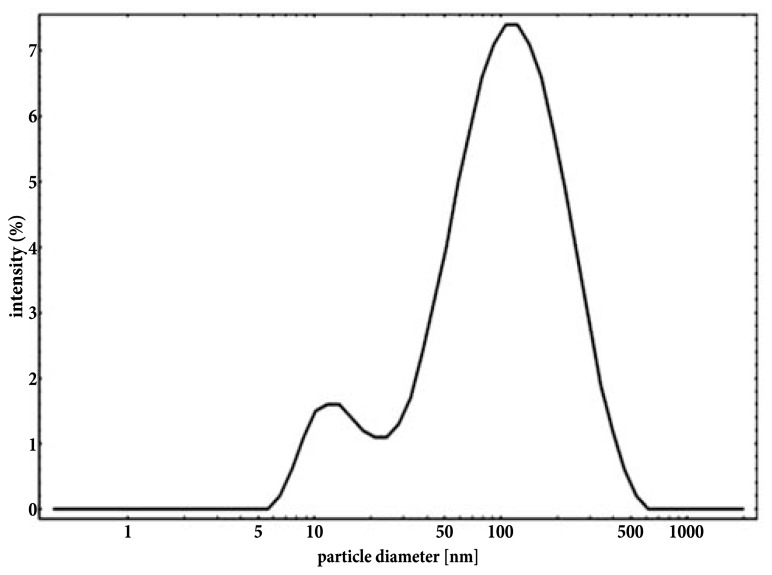
**Intensity-based particle size distribution of an aqueous suspension of NM-300K silver nanoparticles obtained from DLS analysis**.

With the use of transmission electron microscope, AgNPs suspension appeared as a mixture of heterogeneous particles by size and shape (**Figure 1A**). Most particles were spherical, but the sporadic presence of regular polygonal particles was also observed. Particle size distribution (obtained from 8872 particles) showed two major populations, with median (±MAD) diameters of 6(±2) and 18(±2) nm (with first and third quartiles at 5–8 and 16–19 nm, respectively; **Figure 1B**). Particle size distribution from DLS data agreed with TEM data showing a polydisperse sample (PDI = 0.485) with particles having diameters between 5 and >500 nm with the most frequent population having diameters between 6 and 20 nm (**Figure 2**). No differences were observed in the AgNPs size and shape after incubation in the presence of the culture medium MHB with no aggregates identified at any time (data not shown).

ICP-MS analysis to evaluate the chemical purity of the AgNPs showed that the contaminant elements (V, Al, Fe, Mg, Si) detected in the samples were comparable with those in the blanks. Therefore, these contaminating elements were considered as negligible for the purpose of the present study (data not shown).

### *Salmonella* STRAINS

Bacterial strains were chosen taking into account their importance for public health, as in the case of *S.* Enteritidis and *S.* Hadar (EFSA, 2008) and for their ability to adapt to unfavorable chemical–physical environments, as is the case of *S.* Senftenberg (Ng et al., 1969; Broennum Pedersen et al., 2008). *Salmonella* strains antimicrobial resistances have been described in **Table 1**.

### AgNPs SUSCEPTIBILITY ASSAY

To compare the antibacterial effect of AgNPs on the *Salmonella* serovars and to assess the duration of this effect over time, a cell culturability assay in liquid culture was carried out. Equal numbers (approximately 1 × 10^8^ CFU/m) of CFUs of *S.* Enteritidis, *S.* Hadar, and *S.* Senftenberg were incubated individually with different concentrations of AgNPs or AgNO_3_. At different time points, aliquots were removed and plated onto selective medium to count culturable cells.

**FIGURE 3:**
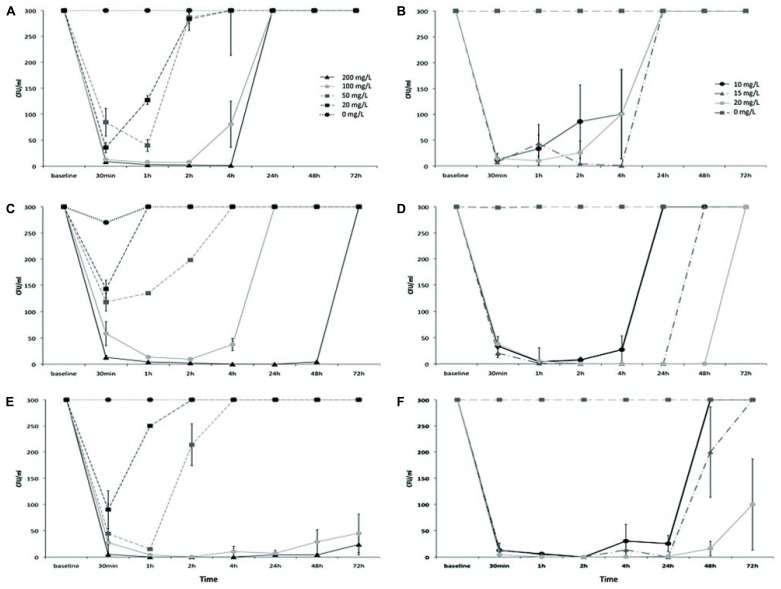
**Growth curves of *Salmonella* Senftenberg (A,B), *Salmonella* Hadar (C,D), and *Salmonella* Enteritidis (E,F), in the presence of different doses of AgNPs (left side) or AgNO_3_ (right side)**.

As shown in **Figure 3**, AgNPs treatment reduced *Salmonella* counts to different extents depending on the serotype. The effect was proportional to the dose with 200 mg/l being the most effective, and 20 mg/l the least effective for all tested *Salmonella* serovars. Moreover, a strong difference in the recovery from the antibacterial effect was observed as *S.* Enteritidis cells never recovered, even after 72 h of incubation with the maximum AgNPs tested concentration (**Figure 3E**). *S.* Hadar clearly displayed a total recovery at 72 h (**Figure 3C**) while *S.* Senftenberg was inhibited by AgNPs only for 4 h (**Figure 3A**). When *Salmonella* strains were incubated in the presence of the minimum concentration of AgNPs (20 mg/l), their recovery was variable, as AgNPs inhibited *S.* Hadar to a lesser extent than *S.* Enteritidis and *S.* Senftenberg (**Figures 3A,C,E**).

However, strong differences in the recovery patterns were observed between the three tested serovars. As shown in **Figure 3**, AgNPs were the most effective against *S.* Enteritidis (**Figure 3E**), with a noticeable reduction in bacterial numbers after just 1 h of exposure to a dose of 50 mg/l. In the presence of 100 mg/l of AgNPs the inhibition continued after 72 h of exposure. In the case of *S.* Hadar (**Figure 3C**), although AgNPs displayed effectiveness at the same doses that were effective for *S.* Enteritidis, complete recovery from the antibacterial effects of AgNPs was observed between 1 and 72 h of exposure, depending on the dose. AgNPs were not effective against *S.* Senftenberg (**Figure 3A**), which started to grow between 1 and 4 h of exposure to AgNPs, depending on the dose.

A comparable dose-effect and a parallel trend of reduction in bacterial counts and recovery from the antibacterial effect was observed in the presence of AgNO_3_ but with one order of magnitude of difference, for each of the tested *Salmonella* serovars (**Figures 3B,D,F**).

### AgNPs RESISTANCE OF *Salmonella* STRAINS

The selective loss of antimicrobial effectiveness, depending on the *Salmonella* serovar, consented to hypothesize the presence of a specific mechanism of resistance.

**FIGURE 4:**
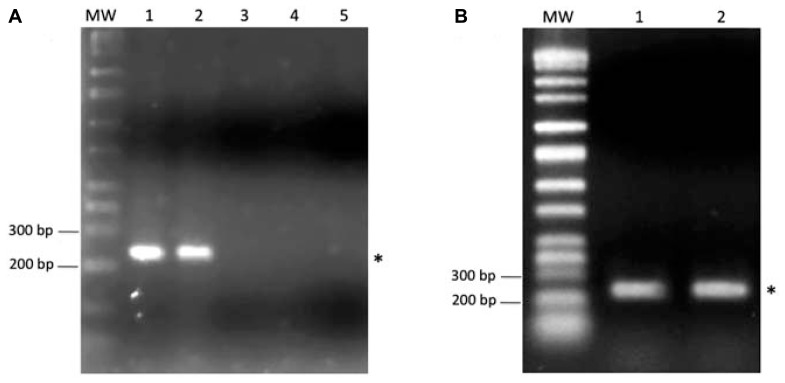
**(A)** One percent agarose gel showing the PCR product of SilB amplification from total DNA of *Klebsiella pneumoniae*, clone ST258 (lane 1), *Salmonella* Senftenberg (lane 2), *Salmonella* Hadar (lane 3), *Salmonella* Enteritidis (lane 4). In the lane 5 was run the negative control (DNA template replaced by sterile water). **(B)** One percent agarose gel showing the PCR product of SilB amplification from the plasmidic DNA fraction of *Klebsiella pneumoniae*, clone ST258 (lane 1) and *Salmonella* Senftenberg (lane 2). The asterisks indicate the 233 bp PCR product expected for SilB fragment amplification. MW indicates the Perfect DNA Markers (EMD, Millipore, USA), 0.05–10 kb ladder.

To test this hypothesis the three *Salmonella* serotypes, Senftenberg, Hadar, and Enteritidis, were screened for the presence of the SilB gene, which is one of the most plausible resistant determinants found to be involved in both silver and copper resistance pathway of many Gram-negative bacteria (Silver and Phung, 2005) and can be of either plasmidic or cromosomic origin (Silver, 2003; Silver and Phung, 2005). As shown in **Figure 4A**, one PCR product of the size of 233 bp, expected for SilB fragment amplification, was amplified from total DNA extract only in the case of *S.* Senftenberg (**Figure 4A**, lane 2). No amplification products were observed in the case of the other investigated *Salmonella* serovars (**Figure 4A**, lanes 3 and 4) and this is consistent with the fact that only *S.* Senftenberg showed a silver resistant phenotype in the culturability assay. *K. pneumoniae* strain, clone ST258, used as positive control (**Figure 4A**, lane 1), was previously described as carrying the pKPN-IT (JN233704) plasmid, highly related to plasmid pKPN3 identified in *K. pneumoniae* and conferring resistance to arsenic, copper, and silver (García-Fernández et al., 2012).

To verify the plasmidic origin of the investigated SilB gene, plasmidic DNA was isolated and screened by PCR for the presence of SilB gene. The result, shown in **Figure 4B**, clearly indicates that the resistance gene was positioned on the plasmidic portion of DNA in the case of *S.* Senftenberg (**Figure 4B**, lane 2) as it was for *K. pneumoniae* strain, clone ST258, used as positive control (**Figure 4B**, lane 1).

**FIGURE 5:**
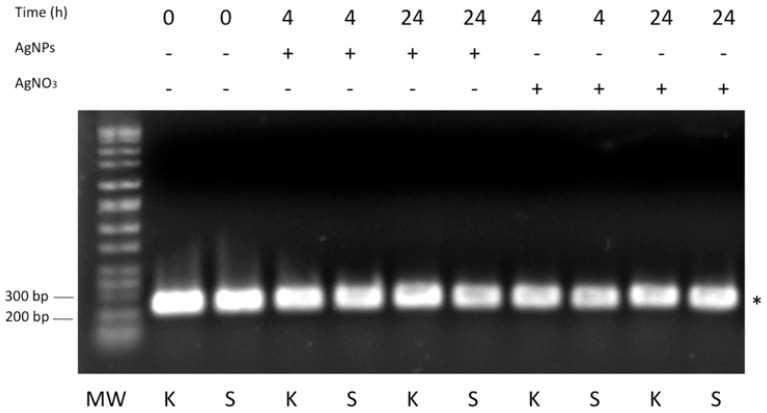
**RT-PCR analysis.** One percent agarose gel showing DNA bands originating from PCR amplification experiments referring to *Klebsiella pneumoniae*, clone ST258 (K) and *Salmonella* Senftenberg (S). The asterisk indicates the 233 bp PCR product expected for SilB fragment amplification. MW indicates the Perfect DNA Markers (EMD, Millipore, USA), 0.05–10 kb ladder.

In order to test the expression of SilB gene in the resistant *S.* Senftenberg strain, in presence and absence of AgNPs or AgNO_3_, qualitative RT-PCR experiments from total RNA extract were carried out. *K. pneumoniae* strain, clone ST258, was used as control. As indicated in **Figure 5**, SilB is constitutively expressed by *S.* Senftenberg strain both in the presence of AgNPs or AgNO_3_ and in the absence of any silver forms with *K. pneumoniae* strain displaying the same expression profile (**Figure 5**).

## DISCUSSION

The continued evolution of bacteria strains displaying resistance to multiple antibiotics, has prompted the scientific community as well as the food business operators to look for antimicrobial alternatives. Thus silver, which is commonly considered a broad-spectrum antimicrobial agent, could represent a suitable option, especially in its nanoform (Silver et al., 2006). There is circumstantial evidence that these particles have good antimicrobial activity; however, the exact way in which they exert this activity is still speculative (Pelgrift and Friedman, 2013). AgNPs have shown antimicrobial activity against a wide array of microbes, probably due to their multiple mechanisms of antimicrobial action, including activity against drug-resistant bacteria, fungi, and viruses (Sondi and Salopek-Sondi, 2004; Pal et al., 2007; Choi et al., 2008; Fayaz et al., 2010; Lara et al., 2010; Samberg et al., 2011; You et al., 2011). Nonetheless the present study demonstrates that AgNPs can be effective as an antimicrobial even in the case of *Salmonella*, but that its success is strongly *Salmonella* strain-dependent, since great differences in terms of effective dose and time of action were observed for the three investigated serovars (*S.* Enteritidis, *S.* Hadar, and *S.* Senftenberg). This is probably due to many factors strictly related to the genetic features of each strain, including the presence of specific genetic determinants of resistance, as demonstrated in the case of *S.* Senftenberg, which specifically expressed the resistance gene SilB. The antimicrobial resistances for the tested antibiotics, displayed in **Table 1**, do not followed the same pattern of silver resistance since *S.* Hadar appeared in general more resistant than *S.* Senftenberg. Silver resistance has been reported in bacteria isolated from both clinical (Carr and Rosenkranz, 1975; Annear et al., 1976; Hendry and Stewart, 1979; Markowitz et al., 1983; Klasen, 2000) and environmental (Belly and Kydd, 1982; Haefeli et al., 1984; Grewal and Tiwari, 1990; Choudhury and Kumar, 1998) settings. The resistance mechanisms include altered cell membranes that decrease uptake of Ag and efflux pumps that pump Ag out of the cell (Silver and Phung, 2005). Among the determinants of silver resistance, SilB was originally found on *Salmonella* plasmid pMG101 (Mchugh et al., 1975) and encodes resistances to Ag^+^, Hg^2^^+^, and tellurite, as well as to several antibiotics (Mchugh et al., 1975; Gupta et al., 1999). *Salmonella* and *Escherichia coli* have, in addition, a related chromosomal Ag^+^ resistance determinant (Gupta et al., 2001; Franke et al., 2003; Silver, 2003).

However, silver export from Gram-negative bacterial cells might also be determined by other efflux systems, as the Agr and CusCBA protein complexes (Franke et al., 2003) that can bind both copper and silver. Moreover P-type ATPases and cation diffusion facilitators that export surplus metals from the cytoplasm can be involved in metal resistance. These determinants are not only necessary for resistance to toxic metal ions such, but also needed to tolerate at high concentrations some metals that are essential at low concentrations (Nies and Herzberg, 2013) as in the case of *Salmonella* spp. CopA and GolT, P-type ATPases (Espariz et al., 2007; Pontel et al., 2007; Osman et al., 2010, 2013).

The silver resistance conferred by plasmid pMG101 contains nine genes (Gupta et al., 1999; Silver, 2003) coding for one efflux ATPase (SilP), two metal-binding proteins (SilF and SilE), and one cation/proton antiporter (SilCBA; Gupta et al., 1999; Silver et al., 2006). All these proteins are supposed to work in a synergistic way as SilP release Ag^+^ in the periplasmic space, SilF functions as a Ag^+^ carrier from its periplasmic site of release by SilP to the periplasmic site of uptake by SilA as part of the SilCBA complex, which drives Ag^+^ out from the bacterial cell (Silver et al., 2006). Within the SilCBA complex, SilA is an inner membrane cation pump protein, SilC is an outer membrane protein and SilB is a periplasmic membrane fusion protein that spans the inner and outer membrane and contacts both SilA and SilC (Gupta et al., 1999; Silver et al., 2006). Due to its central role in the unique Ag^+^ external efflux system, SilB could represent a good marker for the presence of the silver resistance pathway. Since silver has a broad range of actions on cellular processes, silver resistance does not seem to spread as antibiotic resistance does, and which, in contrast, specifically targets one process. In spite of this, plasmid-encoded silver resistance is a matter of concern, since plasmid-mediated metallic salt resistance has been associated with co-resistance to chemotherapeutic antibiotics (Mchugh et al., 1975; Gupta et al., 1999), and furthermore, the fact that it is a plasmid-mediated resistance means it has potential to easily spread among the bacterial community, both commensal and pathogenic (Szmolka and Nagy, 2013). In addition, silver resistance might persist in the clinical setting (Gupta et al., 2001) posing serious threats in consideration of the widespread use of silver in hospital devices, environmental surfaces and disinfection products.

Silver-resistance genes have been assumed to be expressed in response to long-term exposure to Ag, while expression should be lost in the absence of Ag (Lara et al., 2010; Knetsch and Koole, 2011). This could suggest a decrease of evolutionary fitness in bacteria carrying silver resistance genes, due to the genes being permanently carried and copied, but expressed only in the presence of Ag (Pelgrift and Friedman, 2013). However, gene expression experiments reported in this study clearly demonstrated the constitutive expression of SilB gene, both in the presence and in the absence of silver, in both its tested forms: “nano” or ionic. Moreover, *S.* Senftenberg tested in this study was isolated from samples belonging to the food chain where silver is not common as contaminant and only trace levels are expected to be present (Millour et al., 2012).

## CONCLUSION

The results obtained from the AgNPs susceptibility assays demonstrate that AgNPs were effective as antibacterial against all treated *Salmonella* strains only over a short period of time, thus suggesting that the pathogen’s ability to survive in the presence of AgNPs could be a challenge for the proposed application.

Moreover the presence of the plasmid containing the SilB gene in the resistant strain *S.* Senftenberg entails that it could be acquired under environmental selective pressure and transferred to other bacterial species. This is of particular importance in the field of food safety because silver compounds are used to sanitize food production plants in order to eliminate pathogens that are considered to be relevant for public health, as the three investigated *Salmonella* serovars.

Furthermore, the greater activity of AgNO_3_ compared to that of AgNPs suggests that ions are the more effective elements exerting antibacterial activity. However, further evidence is needed to support this hypothesis.

Nevertheless, it is important to emphasize that the present study took into consideration only one type of AgNP; thus the results here discussed cannot be completely generalized due to the broad variability of NP biological activities that depend not only on their chemical form but also on the specific shape and dimensional range. Smaller sized AgNPs (<10 nm) were demonstrated to have higher antimicrobial activity than larger particles (Xu et al., 2004; Gogoi et al., 2006). In addition, Pal et al. (2007), demonstrated that triangular shaped particles of silver were more active as antimicrobials than rods and spherical particles (Pal et al., 2007) and in a study by Lok et al. (2007) oxidized particles were shown to exert different antimicrobial properties (Lok et al., 2007). This indicates that the surface area of the particles is important for their activity, as Ag^+^ release, the determining factor for antimicrobial activity, might be dependent on the surface area. Therefore, the inhibitory or antibacterial effects AgNPs have on *Salmonella* must be evaluated a case-by-case basis in the future.

## Conflict of Interest Statement

The authors declare that the research was conducted in the absence of any commercial or financial relationships that could be construed as a potential conflict of interest.
